# Discovering time-lagged rules from microarray data using gene profile classifiers

**DOI:** 10.1186/1471-2105-12-123

**Published:** 2011-04-27

**Authors:** Cristian A Gallo, Jessica A Carballido, Ignacio Ponzoni

**Affiliations:** 1 Laboratorio de Investigación y Desarrollo en Computación Científica (LIDeCC), Departamento de Ciencias e Ingeniería de la Computación, Universidad Nacional del Sur, Av. Alem 1253, 8000, Bahía Blanca, Argentina; 2 Planta Piloto de Ingeniería Química (PLAPIQUI) - UNS - CONICET, Complejo CRIBABB, Co. La Carrindanga km.7, CC 717, Bahía Blanca, Argentina

## Abstract

**Background:**

Gene regulatory networks have an essential role in every process of life. In this regard, the amount of genome-wide time series data is becoming increasingly available, providing the opportunity to discover the time-delayed gene regulatory networks that govern the majority of these molecular processes.

**Results:**

This paper aims at reconstructing gene regulatory networks from multiple genome-wide microarray time series datasets. In this sense, a new model-free algorithm called GRNCOP2 (**G**ene **R**egulatory **N**etwork inference by **C**ombinatorial **OP**timization 2), which is a significant evolution of the GRNCOP algorithm, was developed using combinatorial optimization of gene profile classifiers. The method is capable of inferring potential time-delay relationships with any span of time between genes from various time series datasets given as input. The proposed algorithm was applied to time series data composed of twenty yeast genes that are highly relevant for the cell-cycle study, and the results were compared against several related approaches. The outcomes have shown that GRNCOP2 outperforms the contrasted methods in terms of the proposed metrics, and that the results are consistent with previous biological knowledge. Additionally, a genome-wide study on multiple publicly available time series data was performed. In this case, the experimentation has exhibited the soundness and scalability of the new method which inferred highly-related statistically-significant gene associations.

**Conclusions:**

A novel method for inferring time-delayed gene regulatory networks from genome-wide time series datasets is proposed in this paper. The method was carefully validated with several publicly available data sets. The results have demonstrated that the algorithm constitutes a usable model-free approach capable of predicting meaningful relationships between genes, revealing the time-trends of gene regulation.

## Background

The genome encodes thousands of genes whose products enable cell survival and numerous cellular functions. The amount and the temporal pattern in which these products appear in the cell are crucial to the processes of life. Gene Regulatory Networks (GRNs) govern the levels of these gene products. A GRN is the collection of molecular species and their interactions, which together control gene product abundance [1,2]. Numerous cellular processes are affected by regulatory networks.

Innovations in experimental methods have enabled large scale studies that allow parallel genome-wide gene expression measurements of the products of thousands of genes at a given time, under a given set of conditions and for several cells/tissues of interest. This technology, called DNA microarray, introduces a variety of data analysis issues (due to the large amount of information to analyze) that are not present in traditional molecular biology [3].

Over the past few years, several statistical and artificial intelligence techniques have been proposed to carry out the reverse engineering of GRNs from monitoring and analyzing gene expression data [1-6]. These techniques vary from the simplest Boolean models to Continuous and Single Molecule Level models [2]. In this regard, model-free approaches are decidedly attractive because of the complexities of dynamic molecular networks [7]. Moreover, most of gene networks are hard to be mapped precisely by any parsimonious mathematical model. Then, data mining approaches offer a way to identify regulatory mechanisms directly from the input/output data without any underlying model. In particular, rule-based approaches offer several advantages when data-driven analysis is performed. They are highly abstract model-free techniques and hence, they require the least amount of data, with an important ability to perform inferences [2]. Additionally, the simplicity of these approaches allows the inference of large size models with a higher speed of analysis. On the other hand, they can merely display qualitative dynamic behavior [2].

Another important aspect to be considered, when dealing with this biological problem, is constituted by the manner in which the temporal patterns of a GRN are captured. As it was mentioned in some other studies [8,9], the time-delayed gene regulation is a common phenomenon. Thereby, multiple time-delayed gene regulations can be considered the norm, while single time-delayed associations the exception [7]. This issue of the time-delayed gene regulations is well recognized by several authors [7,10-13], although, in most cases, they merely deal with the gene networks delayed for one unit of time due to the inherent complexity and computational cost involved.

In this paper, a new machine-learning approach for the inference of time-lagged rules from time series gene expression data is assessed. The discovered relationships, that represent potential interactions between genes, may be used to predict the gene expression states of a gene in terms of the gene expression values of other genes and, in this way, a putative GRN may then be reconstructed by applying and combining these rules. The approach offers several relevant and distinguishing features in relation to most of the existing methods. First of all, the gene expression value discretization criterion performed in this work is neither arbitrary nor uniform. Secondly, it can infer rules with multiple time-delays. Also, the results can be easily interpreted since the rules are derived from schemes that classify the different regulation states. As well, the algorithm can infer the relationships between genes automatically from multiple microarray time series data. Finally, the new method is capable of processing large scale datasets in order to perform genome-wide studies.

The rest of the paper is organized as follows: in the next subsection, several machine learning techniques available in the literature for GRN inference are overviewed. Following, the underlying methodology and the main characteristics of the new algorithm are presented. Next, two experimental phases are described. The first one is constituted by a detailed comparison with several related methods; the second one contains a performance analysis of the method in a genome-wide scale. Finally, some conclusions are put forward.

### Related work

As it was aforementioned, several statistical and artificial intelligence techniques have been proposed in order to reconstruct a GRN from gene expression data. In this section, some of the approaches from the area of machine learning will be summarized. For a more detailed review please refer to [2,3,6].

Clustering techniques are one of the most used computational strategies for analyzing microarrays [14-16]. These approaches approximate regulatory networks by identifying groups of co-expressed genes and by analyzing relationships between their regulatory regions and DNA binding motifs targeted by known transcription factors. However, determining the interactions that can exist between different genes is not easily achieved by direct clustering, particularly because genes can participate in more than one gene network. Another limitation of these approaches is that they assume co-expression is always equivalent to regulation. Moreover, these methods imply symmetric relationships between the genes, which might not always correspond to biological phenomena [10].

Bayesian Networks also constitute the basis of several approaches for GRN inference [11,17,18]. These methods employ conditional probabilistic distributions for gene interaction modeling. Particularly, Friedman *et al. *[17] proposed a heuristic algorithm to produce networks which appeared biologically plausible for the yeast cell cycling array data. As another example, in Zou and Conzen [18] a model for genetic regulatory interactions that combines the simple Boolean logic semantics of Boolean Networks and the uncertainty offered by Bayesian Networks was proposed. Despite the strong theoretical rationale behind these approaches, the exponential explosion of the parameter space required for these models, together with the large quantity of data needed to make reliable inferences, reduces their capacity to infer complex GRNs by only using gene expression data. Moreover, since they are acyclic directed graphs, they cannot represent an auto regulation or a time-course regulation in a straightforward way [19].

As well, the Apriori Algorithm is also a classic method, designed to operate on databases for learning association rules [20]. In Baralis *et al. *[21], this data mining technique was used for the extraction of time-delayed association rules in gene expression data. They mine the rules by means of the application of the algorithm on matrices of time-lagged gene expression profiles, similar to those used in [7]. Following the same basis, in Nam *et al. *[22] a modified version of the Apriori Algorithm was proposed. In this work, they extended the original method in order to consider temporal item sets, allowing the extraction of temporal association rules. Since the performance of this method highly depends on the parameters set being selected, they employ a parameter fitting phase that uses known regulation information in order to find the best setup for a given dataset. However, Apriori based methods also scale poorly and are sometimes impractical with high dense datasets such as microarrays [3], due to the high-computational cost of the evaluation of candidate and test sets.

Decision trees are also among the most popular classification algorithms in current use within data mining and machine learning research areas. In this sense, Soinov *et al. *[12] approached the task of reconstructing GRNs as a classification problem, proposing the application of decision trees to infer classifiers that may represent regulatory rules (relationships) between genes. In this work the authors have considered at most one unit of time-delay and have applied the C4.5 algorithm to infer the decision trees [23]. In the same regard, Li *et al. *[7] proposed the application of decision trees to infer relationships with one or more units of time-delay, as a generalization of Soinov's method. For each target gene, they constructed its time-delayed gene expression profile and then used a decision tree to discover the time-delayed regulations that modulate the activities of the target gene. Although these are sound and conceptually interesting approaches, working directly on large datasets of thousands of genes, they can be computationally highly demanding.

Boolean Networks were one of the first models to be employed in GRNs inference [24,25] and new variations of this approach have been recently published [26]. These models basically aim at inferring logical rules from a discretization of gene expression time series. Even though these models can be easily applied, they depend on arbitrary discretizations of the gene expression values [12], which impose strong assumptions and restrictions about the biological system under study. In order to overcome this limitation, Ponzoni *et al. *[10] proposed a machine-learning algorithm called GRNCOP based on combinatorial optimization that does not assume arbitrary nor uniform gene expression value discretizations. The thresholds are calculated dynamically by applying the same continuous-valued attribute discretization techniques as those used for classification algorithms based on decision trees. However, this discretization is performed merely for regulatory genes, since the thresholds for the target genes are calculated by using the mean expression value. Another limitation is that it is only able to infer rules of one unit of time-delay at most. This method is in fact the antecessor of the approach proposed in this work, called GRNCOP2, in which all these limitations are overcome.

With the exception of clustering approaches, all of the aforementioned rule-based mining algorithms have been assessed only for highly reduced datasets. Although performing over a small amount of data can give an idea of the performance of a method, in any real scenario the large size and the amount of datasets available impose another challenge on the reconstruction of GRNs that few authors have considered: the scalability problem [3]. This issue represents one of the most important weaknesses of the previously cited studies for rule-based inference methods, due to the lack of evidence that they can actually perform over large datasets, thus preventing their applicability in any complex study. In this context, the algorithm presented in this work exhibits most of the desirable features mentioned before, and in addition it successfully deals with the main drawbacks detected in the existing methods.

## Results and Discussion

For this work, the time series encoded in the gene expression dataset are represented by means of a gene expression data matrix, ***X***, where the rows and columns represent genes and time-points, respectively. In this way, each element ***x**_ij _*of ***X ***contains the expression value of gene*_i _*in the time-point (sample or experimental condition) *j*. Although the gene expression values belong to a continuous range of the real numbers, it is possible to define a finite expression state set for each gene by means of a discretization procedure. Such a procedure is required in order to encode the inputs for any combinatorial optimization process or machine-learning method. In this paper we work with two states for each gene: *upregulated *(when the gene is expressed with a value greater than a specific discretization threshold) and *downregulated *(when the gene is expressed with a value lower or equal to a specific discretization threshold).

Therefore, the inference process requires the definition of discretization thresholds in order to infer putative regulatory relationships between genes. These "discretization thresholds" have traditionally been estimated as unique static values for all of the genes under study. For example, *ad hoc *methods based on mean expression values have been applied. However, a more biologically meaningful scheme should model the fact that a gene may actually have distinct discretization thresholds in relation to different genes in the GRN [10]. For example, regarding the regulatory network under study in this work that corresponds to the *Saccharomyces cerevisiae *organism, the *CLB2 *and *SWI5 *genes are shown to be potentially activated by *CLB1 *gene, but their respective upregulation thresholds are different. Therefore, a fundamental problem consists in the estimation of the regulation thresholds for each gene in relation to every potential target gene, which can reflect significant interactions between them with a higher level of accuracy.

In this regard, two different types of discretizations are defined in this paper. Broadly, the first one is to set the state of each target gene, and it is called Target Discretization Threshold (TDT). The second one is to evaluate the potential interaction between each pair of genes and it is calculated in an adaptive gene-pair-specific way. This last discretization is called Relative Regulation Threshold (RRT).

At this point, our hypothesis is stated as follows: rules - potential regulatory relationships - may be accurately inferred from gene time series data to reveal how the present and future state of a gene may be affected by the gene expression values of the other genes, taking into account their RRT. In this paper, we consider time-lagged rules that represent the situation in which the state of a gene*_i _*in a time-point *j *depends on the gene expression values of other genes in the previous time-point (that is to say, previous experimental condition) *j *- *w*, where *w *is a non-negative integer value representing the time-delay in the relation. The syntax of the rules is: < symbol >< gene*_r_*>*w*→< symbol >< gene*_i_*> where gene*_r _*and gene*_i _*stand for gene regulator and gene target respectively. The symbol + (-) on the left side of the rule indicates above (below) some RRT for the gene*_r _*w.r.t. gene*_i_*, whereas the symbol + (-) on the right side of the rule indicates *upregulated *(*downregulated*) state, depending on the TDT for the gene*_i_*. For example, the rule +/- *CLB1 3*→ +/- *CLB5 *denotes that, if *CLB1 *is above its RRT in relation to *CLB5, t*_CLB1,CLB5_, in a time-point *j*, then *CLB5 *will be *upregulated *in the time-point *j*+3 and, if *CLB1 *is below or equal to *t_CLB1,CLB5_*in a sample *j*, then *CLB5 *will be *downregulated *in the sample *j*+3. The types of rules obtained through this scheme are similar to those studied in [7,10-12]. The main difference is that this scheme allows the representation of both simultaneous and time-lagged rules spanned in any unit of time-interval, which constitutes the kind of rules that GNRCOP2 is capable of inferring.

GRNCOP2 infers the association rules described above by exploring the possible combinations of interactions between each pair of genes. In this sense, six particular cases are assumed, which are represented by the non null integer numbers between -3 and 3, and a special case that indicates the absence of any relation represented by the number 0. All of these cases are described in Table 1.

**Table 1:** Types of rules inferred by GRNCOP2

Rule type	Time-lagged rule associated
-3	+ gene*_r _w*→ - gene*_i_*
-2	- gene*_r _w*→ + gene*_i_*
-1	+/- gene*_r _w*→ -/+ gene*_i_*
0	gene*_r _*does not interact with gene*_i_*
1	+/- gene*_r _w*→ +/- gene*_i_*
2	+ gene*_r _w*→ + gene*_i_*
3	- gene*_r _w*→ - gene*_i_*

Summary of the different types of rules inferred by GRNCOP2, where *w *denotes the time-delay in the regulation.

In mathematical terms, the inference of the rules to reconstruct a GRN can be expressed as the following combinatorial optimization problem:(1)

subject to:

• *n *is the number of genes in the microarray dataset.

• *m *is the number of time-points in the microarray dataset.

• ***X ***∈ ℜ^*n*x*m *^is the matrix with the expression data.

• ***P ***is the space of all vectors *v *of dimension *n *such that *v(r) *∈ {-3, -2, -1, 0, 1, 2, 3} ∀ *r, r *= 1..*n*.

• δ (***X**, i*) is the discretization function such that δ (***X**, i*) = *D_i _*and *D_i _*∈ {-1,1}^*n*x*m*^.

•  ∈ ***P ***is a classifier for *D_i_*.

• σ*(, *D_i_*) is a general performance function of  as a classifier of *D_i_*.

From now on, the symbol ∏*^w ^*indicates the set of optimal classifiers, ∏*^w ^*= {, , ..., }, for a given time-delay *w*. It is important to note that the general optimization problem is the same for all the time-lagged rules. The only difference lies in the definition of the discretization function *δ (**X**, i)*, because the delayed rules are based on expression value discretizations of ***X ***that consider the required temporal shift.

## Algorithm

**Figure 1:**
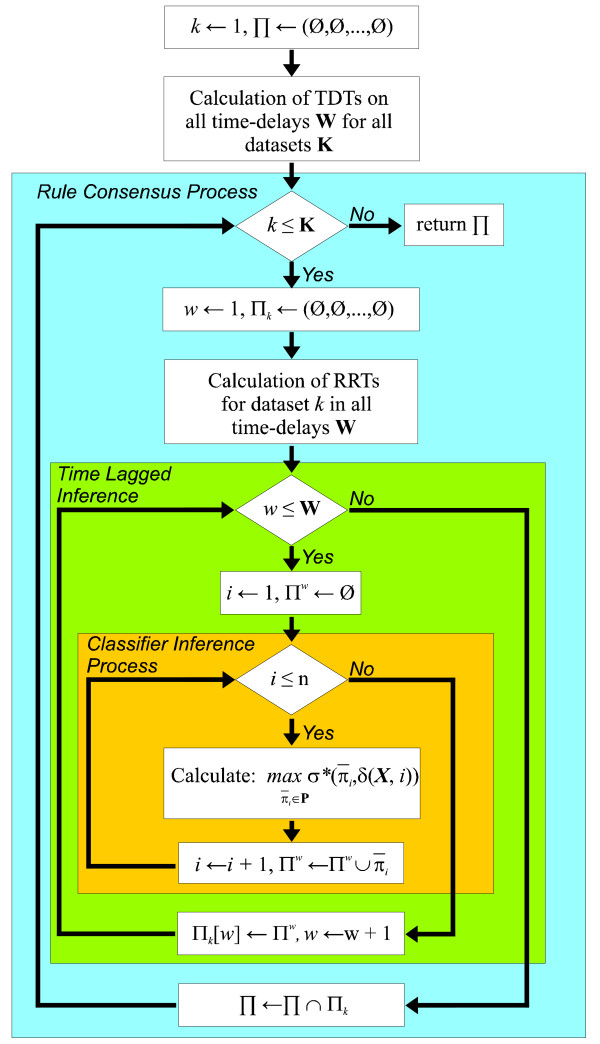
**General schema of the GRNCOP2 Algorithm**.

Although the basic ideas behind GRNCOP, more specifically the adaptive regulation thresholds and the combinatorial optimization of rules classifiers, remain in GRNCOP2, the new method constitutes a significant evolution of the previous algorithm due to the challenges that impose the improvements being proposed. Figure 1 shows an abstract representation of the approach. The machine-learning process used to obtain the rules iteratively performs the search through all the datasets for all the required time-delays. The algorithm receives as input a set of **K **microarray time series datasets and returns an array **П **of dimension **W **that contains, in each position *w*, the set of rules obtained with a time-delay *w*. The following subsection will explain in detail the main characteristics of the procedure.

### An improved discretization technique for the target genes

In order to obtain the TDTs of the target genes, GRNCOP2 employs a technique that is able to infer the gene states in a more precise way, compared to the mean expression value used in GRNCOP. In mathematical terms, the procedure for the discretization of a gene*_i _*can be defined as follows:(2)

subject to:

• *S *is the set of sample values for the gene*_i_*.

• *S_1 _*∩ *S_2 _*= ∅, *S_1 _*∪ *S_2 _*= *S*, |*S_1_*|>1 and |*S_2_*|>1.

• var(*S_1_*) and var(*S_2_*) are the variance of *S_1 _*and *S_2 _*respectively.

• *S_1 _*and *S_2 _*represent the two expression states for the gene*_i_*.

Basically, the procedure divides the samples of the gene*_i _*in the two sets that have the minimum sum of its variances. The cardinality of *S_1 _*and *S_2 _*is required to be greater than one in order to avoid the effects of a possible outlier in the samples, since it is improbable that a gene is clearly expressed or inhibited in only one time-point. Thus, when the samples of a gene*_i _*are separated in a partition that violates this restriction, the gene*_i _*is no longer considered in the inference process for the actual datasets. Another approach could have been to exclude the conflictive time-point in the search of the partition. However, this can lead to the same situation described before, thus reintroducing the issue that was supposed to be fixed.

This technique is in essence a clustering procedure similar to a k-means with k = 2. However, since the number of clusters is 2 and the elements of *S *have a total ordering, the problem can be optimally and efficiently solved through the following deterministic procedure:

• Sort the elements of *S *on an array *L*.

• Search for the element *e *such that var(*L*[1..*e*]) + var(*L*[*e*+1 ..|*S*|]) be the minimum.

• Return (*L*[*e*]+*L*[*e*+1])/2 as the TDT of the gene*_i_*.

It is important to state that, according to Figure 1, TDT values are calculated for each dataset separately.

### Rule Consensus Process

In essence, the main loop of the algorithm applies the same inference method to the **K **microarray time series datasets given as input, and then returns the intersection of the results for all of the datasets. The objective of this procedure, incorporated by GRNCOP2, is to automatically assess the rules obtained by the algorithm through different datasets, thus increasing the degree of evidence required for the potential regulatory relationships to be returned. The intersection of the rules obtained from two datasets *k_1 _*and *k_2 _*is defined as follows:(3)

where:

• **W **is the maximum time-delay established by the researcher.

•  and  are the rules obtained from datasets *k_1 _*and *k_2 _*respectively in all time-delays **W**.

•  and  are the rules obtained from datasets *k_1 _*and *k_2 _*respectively with a time-delay *w*.

Basically, the intersection of the results is the intersection of each component, i.e., time-delay *w*, of the set of rules obtained from the **K **datasets. In an ideal scenario, all the time series microarray datasets should have the same sampling rate and then, a direct mapping between the time-delay *w *and the real time-points would be achieved. However, this is far from any real scenario due to the limited availability of replicas for the same experiment. Moreover, the microarray time series datasets might have been obtained under completely different experimental conditions, and then the sample rates of each one may become incomparable due to possible delays in the regulation process introduced by these experimental conditions. Nonetheless, this type of consensus process for the rules was performed manually by other authors [7,10,12] without any kind of resampling or integration of the data. This leads to the interpretation of a time-delay *w *as an abstract unit that denotes a possible future relationship between the genes participating in the rule, introducing the notion of before, equal and after time, but not assuming when it will exactly occur.

This type of consensus process does not limit the number of microarray datasets employed in the inference process. Thus, the following question might arise: is it necessary to assess the rules in all the microarray datasets? And a straightforward answer is no. Thereby, we have introduced a parameter on the consensus process, called *Rule Consensus Accuracy (RCA)*, which specifies the minimum proportion of datasets in which a rule must predict well in order to be returned by the algorithm as a potential relationship. This parameter does not impose any order of importance among the datasets and thus, all of them have the same weight in the consensus process. Thereby, for example, if the algorithm is executed with 10 time series datasets and the *RCA *parameter is set to 0.60, it means that the rules returned by the algorithm predict well in at least any 6 datasets, no matter which ones. In this sense, and in order to set this parameter, the researchers must take into account the number of datasets available and, following the previous example, a question like this should be answered: is it enough evidence of a feasible regulatory relationship for a rule to be supported by at least 6 of 10 (*RCA *= 0.60) datasets? The answer will naturally depend on the biological nature of the experiments and on the criterion of the researcher.

### Relative Regulation Thresholds

During the discretization, the real numbers corresponding to the gene expression values, which are held in matrix ***X***, are mapped to values -1 and 1 using the function *δ (**X**, i)*. The main question at this point is how to define the RRTs for each gene in relation to the others. A traditional approach consists in using the mean expression value of a gene*_r _*over its corresponding sample set of ***X***. This solution is easy to implement, but it represents a strong simplification of the reality because it assumes a unique putative regulation threshold for each gene w.r.t. the others. It is well known that the gene expression value required by gene*_r _*to activate (or inhibit) a gene*_i1 _*is not necessarily the same value required by the same gene*_r _*to activate (or inhibit) a gene*_i2_*. For this reason, a more flexible and dynamic threshold-selection policy that calculates a specific regulation threshold for each pair of genes is applied in GRNCOP2, as it was previously employed in GRNCOP [10].

In essence, GRNCOP2 considers each expression value shown by a gene*_r _*in ***X ***as its potential discretization threshold in relation to an already discretized gene*_i_*. A partition of the sample set of ***X ***into two subsets, namely ***Do ***and ***Up ***(for *downregulated *and *upregulated *respectively) is generated for each gene*_r _*and each candidate threshold *t. **Do ***contains all the samples where the gene*_r _*has an expression value smaller or equal to *t*, whereas ***Up ***contains all the samples where the gene*_r _*has an expression value greater than *t*. Thereby, ***Do ***and ***Up ***represent a partition of the sample set of the gene*_r _*where it has values equal to -1 and 1, respectively, on the basis of *t*. Here, *t *constitutes the candidate discretization regulation threshold for the gene*_r _*in relation to gene*_i_*.

The next step consists in the calculation of the partition entropy, which is a statistical indicator of the quality of a threshold *t *as a discretization value for gene*_r _*w.r.t. the gene*_i_*. To further illustrate this concept, suppose that we are trying to infer the potential regulators of the gene*_i _*(already discretized by using the TDT). Then, for each gene*_r _*(potential regulator of gene*_i_*), GRNCOP2 selects the threshold *t*, as the RRT, that minimizes the partition entropy by using (4). In numerical terms, the partition entropy is 0 when all the samples satisfy the same rule type (ideal situation from a predictive viewpoint) and the partition entropy is 1 when the samples belong to both regulation scenarios in equal proportion (50 percent and 50 percent). Then, when the partition entropy value associated with a discretization approximates to 0, the threshold that generates this discretization represents a better solution. Thus, such a threshold value allows to optimally detect potential significant relationships between gene*_i _*and gene*_r _*in terms of the rule type (see Table 1). The entropy calculation is based on definitions given in [27] and the entropies for ***Do ***and ***Up ***are based on the discretized values of the gene*_i _*obtained with the corresponding TDT. The partition entropy equation was previously applied by Kohani [28] as follows:(4)

where:

• *R *identifies the gene under consideration (potential regulator).

• ***t ***is the partition threshold.

• ***X_S _***is the set of samples of gene*_r _*corresponding to the time series ***X***.

• ***Do ***is the subset of ***X ***with the samples where the gene expression value of the gene*_r _*is less than or equal to *t*.

• ***Up ***is the subset of ***X ***with the samples where the gene expression value of the gene*_r _*is greater than *t*.

After that step, for each possible gene*_i_*, the function *δ (**X**, i) *maps the corresponding gene expression values in ***X ***to the discrete matrix *D_i _*using the previously calculated thresholds. Thereby, each gene*_i _*in the original matrix ***X ***is associated with a discrete matrix *D_i_*. However, unlike GRNCOP and due to efficiency reasons, in GRNCOP2 the discrete matrix *D_i _*is not actually calculated. Instead, each element of *D_i _*is computed by demand by means of an indirect access to the global matrix ***X***, through the specific regulation threshold for gene*_r _*with respect to the gene*_i_*, in the corresponding time-delay *w *and dataset *k*, thus improving the computational time required for execution.

### Time-Lagged Inference

The time-lagged inference process is represented as the middle loop in Figure 1. It merely consists in iteratively searching for rules in each time-delay *w *employing the Classifier Inference Process. However, there are several key aspects in this process that are important to remark. For each discrete matrix *D_i_*, the actual time-delay *w *under evaluation needs to be considered, i.e. the temporal shift for the vector encoding the expression values of the gene*_i_*. Thereby, in order to evaluate all the possible regulators for the gene*_i_*, it is necessary to remove of the first *w *time-points of the gene*_i_*, the last *w *time-points of the rest of the genes (those that will act as possible regulators for the gene*_i_*) and subsequently realign the samples. Thus, as the value of *w *increases, fewer are the samples from which the rules can be inferred. This limits the max value of **W **to *m*-4, being *m *the number of time-points of the dataset that has the fewer amount of samples. This limitation is due to the TDTs employed in this paper, since it requires at least 4 samples to determine the states of a gene*_i_*. In the extreme case that **W **is set to the max possible value, the discrete matrix *D_i _*will have only four time-points for that dataset, increasing the possibility of inferring rules by chance due to the low number of samples. Nonetheless, it is a requirement for the researcher to establish the best value for **W**, depending on the amount of time-points of the available datasets and on the likelihood that such events can actually occur. In this regard, suppose that **K **datasets are available with *m_k _*time-points sampled at a Δt*_k _*time interval each. Thereby, if the hypothesis regarding the nature of biological experiments is that the regulatory events may occur with at most a Δt*_H _*time-delay, then **W **can be calculated as follows:(5)

The previous equation is very simple and merely consists in the ratio of the max regulatory delay given by the hypothesis and the minimum time sampling of the datasets, bounding this value to the maximum possible time window for the **K **datasets.

Additionally, the discretization processes also need to consider the time-delay. In the case of the discretization of the target genes, the TDTs are calculated at the beginning of the algorithm, for all time-delays **W **and for all datasets **K**. This implies that the first *w *elements of the samples, particularly when *w *≥ 1, are omitted in the calculation of the TDTs due to the temporal shift needed to infer the time-lagged rules. In the same regard and in the case of the discretization policy employed for the potential regulatory genes, the RRTs are calculated omitting the last *w *elements of the samples. However, it is necessary to calculate the RRTs at the beginning of the Rule Consensus Process in order to reduce the amount of space required for all possible combination of gene*_r_*, gene*_i_*, time-delays *w *and datasets *k*.

### Classifier Inference Process

As defined in (1), the optimization problem consists in finding a set of optimal  which define potential rules between the gene*_i _*and the other genes (potential regulators). Basically,  is a vector that represents the set of potential regulators of the gene*_i_*. Each component *r *of the vector holds an integer value between -3 and 3, which represents one of the seven regulatory cases shown in Table 1. Thus,  (*r*) indicates the regulation case detected between gene*_r _*and gene*_i_*, in other words,  is a gene profile classifier that represents the potential regulators for the gene*_i _*along with the characteristics of these potential relationships.

Taking into account that the rules inferred by GRNCOP2 are pair-wise qualitative, the components of  can be assumed as independent from each other from an optimization point of view. Thus, the optimal classifier corresponding to a discrete matrix *D_i _*can be calculated in a greedy manner by means of a constructive approach, maximizing a performance function on each component (*r*). In our Classifier Inference Process, the optimization process for , as it was introduced in (1), is performed as follows:(6)

where

• *c *∈ {-3,-2,-1,1,2,3} is one of the regulatory cases shown in Table 1.

Note that the definition of σ*(, *D_i_*) is not necessary due to the assumption of independence among the components of . In this work, the following performance function is used for optimize the *r*-th component of :(7)

where

• ***TP_c _***(True Positives for the rule type *c*) is the number of positive cases of *D_i _*correctly classified by (*r*) when it is considered as a rule of type *c*.

• ***FN_c _***(False Negatives for the rule type *c*) is the number of positive cases of *D_i _*incorrectly classified by (*r*) when it is considered as a rule of type *c*.

• ***TN_c _***(True Negatives for the rule type *c*) is the number of negative cases of *D_i _*correctly classified by (*r*) when it is considered as a rule of type *c*.

• ***FP_c _***(False Positives for the rule type *c*) is the number of negative cases of *D_i _*incorrectly classified by (*r*) when it is considered as a rule of type *c*.

In the previous formula, the first factor is the positive predictive value, whereas the second one is the negative predictive value. Both factors generate values between 0 and 1 and consequently σ((*r*), *D_i_, c*) is always in this range. The best scenario for a potential interaction between a gene*_i _*and a gene*_r _*is obtained when σ((*r*), *D_i_, c*) = 1 because this represents the situation where all expression states were correctly classified, whereas σ((*r*), *D_i_, c*) = 0 refers to the opposite case. Note that *c *= 0 is not considered in the maximization of the performance function since the values of ***TP_c_**, **TN_c_**, **FP_c _***and ***FN_c _***cannot be determined in that case. The main difference between this performance function and the formula employed in [10] is that this one is focused on the precision of the rules as defined in Table 1.

In practice, a threshold value (namely the *Accuracy *parameter) is established in order to return the rules that achieve a score above that specific value. This value acts as a cut off for the components of , discarding those rules that do not predict well according to the maximum value of (7). The discarded rules are considered as rules of type 0 according to Table 1. For the cases 1 and -1, the performance function in (7) is applied as stated, differing only in the way that the positive and negative cases are considered. For instances 2, 3, -2 and -3, only one factor of the performance function is employed (the one corresponding to the rule type) and, in order to avoid the rules that perform above the *Accuracy *parameter with a small ***TP (TN) ***(in relation to the number of samples), an additional parameter called *Sample Coverage Percentage *(*SCP*) is defined. This parameter establishes the minimum proportion of ***TP ***(***TN***) that a rule of the cases 2, 3, -2 and -3 needs to achieve in order to be returned by the algorithm. Both parameters (*Accuracy *and *SCP*) where also utilized in the previous version. Since GRNCOP2 automatically assesses the rules in multiple microarray datasets, the accuracy assigned to each rule that suits the consensus process is the minimum value achieved on all datasets, i.e., the most conservative approach.

Regarding the best setting for these parameters, several authors [7,10-12] consider that the confident relationships between genes are those that perform with an accuracy above 0.70. In this sense, and as a rule of thumb, an *Accuracy *parameter of 0.75 should be enough in order to return high quality regulatory relationships between genes in terms of (7). In the case of the *SCP *parameter, the rules of the cases -2, 2, -3 and 3 are more likelihood to be obtained by chance. Thereby, the only way to ensure confident rules of these cases is by means of the set of the *SCP *parameter close to the max value (i.e. 1). If the *SCP *parameter is set to 1, then none rule of these cases is returned by the algorithm. As stated above, GRNCOP2 calculates  using the same constructive approach employed in [10], which explores all possible combinations of values for its components (*r*). To sum up, GRNCOP2 computes the performance function defined in (7) for each possible interaction case value (encoded by values ranging from -3 and 3) and assigns the rule type *c *that maximizes it to  (*r*). After repeating this for each  (*r*), with *r *= 1..*n*, the resulting  is the optimal gene profile classifier. Thereby, for a gene expression dataset of *n *genes and *m *samples, the computational complexity of the Classifier Inference Process is of *O*(*m.n*^2^) in the worst case. If the whole inference algorithm is considered, the time required to infer the time-lagged rules from **K **datasets in **W **time-delays is of *O*(**K**.**W***.m.n*^2^). Although at first glance the algorithm seems to be considerably time consuming in terms of computational complexity, it can be efficiently optimized in order to perform genome-wide studies, as it will be demonstrated in the next sections.

## Testing

Two different goals are devised for the study of the new method's performance. First, it is important to analyze the quality of the results of GRNCOP2 with respect to the previous version [10] and with respect to other related approaches available in the literature [7,11,12]. For this analysis, GRNCOP2 was tested using the same 20 yeast genes selected by [7,10-12] from the microarray data in [16], in order to achieve a fair comparison. However, although performing over these reduced datasets can give an adequate view of the method's performance for the comparison with other approaches, the scalability problem imposes another great challenge [3]. To this end, in a second experimental phase the performance of GRNCOP2 on a genome-wide study for the *Saccharomyces cerevisiae *organism with complete datasets was carried out.

### Performance assessment

In order to measure the quality of the results of a gene rule mining algorithm, the most frequently used technique is the rule-by-rule analysis of the biological relevance of the relationships obtained by the method. This is done by means of a search through the literature, looking into known biological interactions for the genes under consideration. This approach proves to be sound when a single method is evaluated; however, it has drawbacks that make its application in most scenarios almost impossible. First, it is only applicable when a small set of rules is evaluated, since the whole process is performed manually. Another disadvantage is that it can not be used for comparing several methods, since the quality of a rule is biased by the expert that evaluates it, and therefore it is impossible to establish a fair order of merit for the algorithms under consideration. We do not claim that the use of this evaluation process for a gene rule mining method is inadequate; we just say that it needs to be used as a complement of some other technique that allows fast, direct and unbiased evaluation and comparison of different approaches.

In this context, several complex analyses of potential associations between genes are available in different databases for the yeast organism [29-33]. These studies can be used for the automatic assessment of the quality of the results obtained by an algorithm measuring several well-known data mining metrics, such as *precision, sensitivity *and *specificity*. Regarding the Yeastnet v. 2 [29], 102.803 linkages among 5.483 yeast genes were reported as potential gene-pairs associations, assigning a score value for each association (with stronger associations scoring higher). In the same way, the Gene Ontology (GO) annotation [30] is another source of potential associations for genes. In [29], 66.174 reference gene pairs representing all gene pairs sharing any GO biological process terms between levels 2-10 of a Gene Ontology annotation (downloaded from the *Saccharomyces cerevisiae *Genome Database (SGD) [30]) were used as a benchmarking set. Additionally, the co-citation approach [31-33] offers another source of independent information in order to benchmark the results of gene rule mining algorithms. In this case, a set of 29.135 Medline abstracts that included the word "*Saccharomyces cerevisiae*" for perfect matches to either the standardized names or common names (or their synonyms) of 5.794 yeast genes was analyzed in [33]. They report a set of 29.483 gene pairs assigning a score value for each association (with stronger associations scoring higher).

Therefore, the main idea for the evaluation framework of the methods is to measure the *precision, sensitivity *and *specificity *achieved regarding each one of the previously mentioned studies. Additionally, in the case of [29] and [33], the *score *measured as the average score values of the rules found by a method is assessed. However, since this kind of information does not consider either the time-delay in the rules inferred by the methods or the direction of the actual interaction (none of the genes are stated as regulator or target), a convention must be introduced in order to make a fair comparison between the different algorithms. In this regard, the results of an algorithm will be transformed in order to represent the same kind of information of the benchmarking sets, i.e., only the actual set of gene-gene interactions will be considered for the measurement, leaving aside the notions of time-delay and regulator-target of the rules being inferred. This avoids the repeated validation of multiple rules (due to different time-delays or symmetric links) through the same match in the benchmarking sets, a situation that might produce an unfair comparison. Nonetheless, only equal time-delayed inference intervals will be considered during the comparison of the algorithms.

### Comparative study

In this section, the performance of GRNCOP2 will be compared with the functioning of some representative machine learning methods that are presented in the literature. The predictive efficacy was tested using the microarray data in [34], which also includes data from *Saccharomyces cerevisiae *cell cultures [35]. These datasets were synchronized by three different methods: cdc15, cdc28, and alpha-factors, and they were sampled at intervals of 10 min, 10 min and 7 min respectively. Therefore, the corresponding gene expression datasets may be defined as statistically independent [13]. For the analysis performed in this section, the following 20 genes were used in order to agree with the studies in [7,10-12]: *CLN1-3, CLB1-2, CLB4-6, MCM1, SIC1, CDC28, CDC53, MBP1, CDC34, SWI4-6, SKP1, CDC20 *and *HCT1*. Only adjacent equidistant measurements at the same units of time were considered with the aim of facilitating the interpretation of time-delayed rules. In this context, several time points for the dataset cdc15 were truncated resulting in a total of 15, 17 and 18 available time points for the cdc15, cdc28 and alpha-factor datasets respectively.

In this comparative study, the *precision, specificity *and *sensitivity *were calculated regarding the reduced search space determined by the 20 genes. Table 2 shows the characteristics of this search space, which consists of 190 possible gene-gene interactions (where the time-delay rules and the symmetric links are not considered in order to match the type of information of the benchmarking sets). It is important to note that, in this case, the *precision *of the 190 possible combinations of gene-pairs in the reference studies determines the probability of randomly selecting a pair of genes that are validated by these sets. In other words, the values in the *precision *and *score *columns of Table 2 would be the expected values if random sets (uniformly distributed) of gene-pairs were selected. Thereby, it is very important that the algorithms perform above these numbers.

**Table 2:** Characteristics of the 190 possible gene pair-wise interactions

	Yeastnet		Co-citation		GO	number of possible associations
		
	*precision*	*score*	*precision*	*score*	*precision*	
All gene pair-wise combinations	51.58%	1.53033843	43.68%	1.3487118	45.26%	190

Characteristics of the 190 possible gene pair-wise interactions for 20 genes in terms of *precision *and *score *metrics on the Yeastnet, Co-citation and GO reference sets.

Due to differences in the availability of the methods and in the results reported by [7,10-12], this analysis is performed in three different stages. In subsection A, an evaluation of the improvements of GNRCOP2 with respect to the previous version is presented. Subsection B corresponds to the comparison with the other selected methods available in the literature [7,11,12]. Finally, in subsection C, the biological relevance of the rules found by GNRCOP2 for this reduced problem instance is discussed.

#### A. Performance of GRNCOP2 vs. GRNCOP

The analysis of the improvements of GRNCOP2 over GRNCOP [10] was performed for the 20 yeast genes previously mentioned on the cdc15, cdc28 and alpha-factor datasets. In order to perform a fair comparison, both algorithms employ the Rule Consensus Process previously described with the *RCA *parameter set to 1. In this way, several runs of each algorithm were performed varying the *Accuracy *parameter from 0.60 to 0.90 with increments of 0.05, and the *SCP *parameter from 0.60 to 0.95 with increments of 0.05. This result on a total of 56 runs for each method, and the set of associations obtained in each case were measured in terms of the *precision, sensitivity, specificity *and *score *metrics previously defined. The *Accuracy *value of 0.95 was omitted since both algorithms were unable to find any rule with this setting. It is also important to state that the focus was put on simultaneous and single time-delayed rules (i.e. **W **= 1 in the case of GRNCOP2), since GRNCOP was not designed for searching rules with multiple time-delays [10]. The average results of the 56 runs in term of *precision, specificity *and *sensitivity *on Yeasnet [29], GO [30] and Co-citation [33] are shown in Table 3 together with the average *score *in the case of [29,33]. The results of both algorithms in each individual run regarding the previously mentioned metrics are available in the additional file 1.

**Table 3:** Average values for the metrics achieved by GRNCOP2 and GRNCOP

		GRNCOP2	GRNCOP	RANDOM
	average *precision*	**84.50%**	76.69%	51.58%
	average *sensitivity*	16.25%	**28.13%**	-
**Yeastnet**	average *specificity*	**94.66%**	82.43%	-
	average *score*	**2.79**	2.49	1.53033843

	average *precision*	**84.13%**	74.86%	43.68%
**Co-citation**	average *sensitivity*	19.02%	**30.46%**	-
	average *specificity*	**95.28%**	82.76%	-
	average *score*	**2.91**	2.50	1.3487118

	average *precision*	**70.73%**	52.25%	45.26%
**GO**	average *sensitivity*	13.93%	**22.55%**	-
	average *specificity*	**91.48%**	76.60%	-

average number of associations		20.84	**43.73**	-

Average *precision, sensitivity, specificity *and *score *values achieved by GRNCOP2 and GRNCOP over 56 runs. The *precision *and *score *of the random selection is also included. The bolded scores denote the best values.

**Figure 2:**
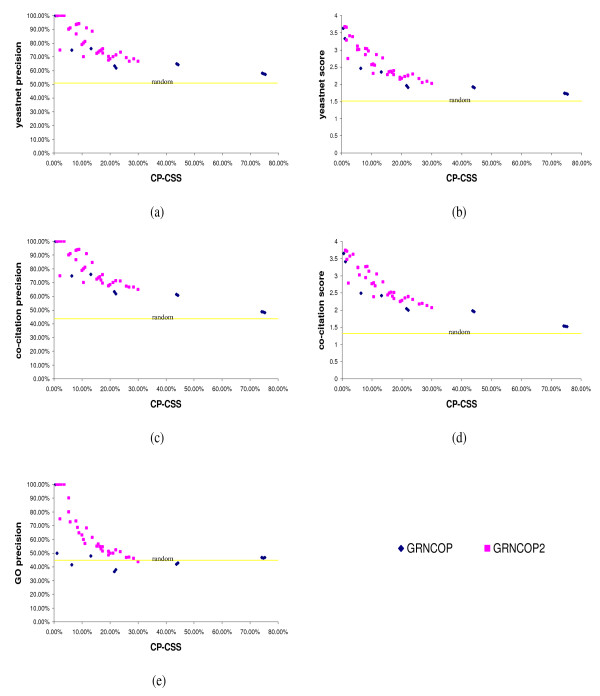
***Precision *and *score *values achieved by GRNCOP2 and GRNCOP**. Values of *precision *and *score *metrics achieved by GRNCOP2 and GRNCOP in each of the 56 runs w.r.t. the *CP-CSS*. Figure 2a: yeastnet *precision*. Figure 2b: yeastnet *score*. Figure 2c: co-citation *precision*. Figure 2d: co-citation *score*. Figure 2e: GO *precision*.

As it can be observed, GRNCOP2 outperforms (on average) GRNCOP in several of the proposed metrics, whereas both algorithms perform significantly above the random selection, as expected. In particular, while GRNCOP2 is on average more precise and more specific than GRNCOP, this last one recovers on average a bigger number of the "relevant interactions" (i.e. it is more sensitive). These results may be explained by the fact that GRNCOP actually recovers on average twice the amount of the associations obtained by GRNCOP2. However, since the values in Table 3 represent the average of the 56 runs, the real picture may be misunderstood. Therefore, in order to correctly establish the behavior of each algorithm, several graphics were performed. Figures 2a to 2e depict the *precision *and *score *metrics achieved by both algorithms in each of the 56 runs w.r.t. the *Coverage Percentage of the Combinatorial Search Space *(namely *CP-CSS*), i.e., the percentage of associations returned by the methods in relation to all possible gene pair-wise combinations (see Table 2.).

Three important observations can be inferred from these figures. The first one is that in various cases, specifically at low values of the *Accuracy *parameter (see additional file 1 for more details), GRNCOP returns almost the 80% of all possible gene pair-wise combinations. In this way, its performance decreases at almost the level of a random selection due to the excessively large amount of associations that are found. This explains the higher values on the average *sensitivity *and *number of associations *showed in Table 3. Moreover, this behavior in not desirable at all since it may limit their applicability in genome-wide contexts, where the number of possible combinations of associations reaches a very high dimensionality. The second but not less important observation is that at the same *number of associations *returned by the algorithms, the interactions found by GRNCOP2 seem (in general) to be more precise and with higher scores than those found by GRNCOP. This is particularly relevant since this behavior evidences the improvements achieved by the modifications included to the inference algorithm previously detailed. The third observation has to do with the different shapes in the distribution of the points of both methods in the figures. Along with the high number of associations discussed above, it seems that GRNCOP has fewer variation in the *precision *and *score *values achieved w.r.t. those obtained by GRNCOP2. However, this can be explained by the fact that GRNCOP is almost insensible to variations of its *SCP *parameter on the values employed in this comparison (see additional file 1). This is most likely related to the mean expression value employed by GRNCOP for the discretization of target genes. In general, an average value of the expression profile of a gene will tend to split the samples into two partitions of approximately the same size (except in the presence of samples with high relative absolute value w.r.t. the others). Thus, only a small number of rules of the cases -3, -2, 2 and 3 will satisfy the *SCP *threshold; in other words, GRNCOP requires even lower values of the *SCP *parameter in order to obtain more rules of these types. Moreover, this situation increases the probability of finding by chance these kinds of rules, given that there are fewer samples for the inference process. Nonetheless, these observations do not invalidate the conclusions regarding the improvements of GRNCOP2 over GRNCOP, since it has been observed that, at lower values of the *SCP *parameters, both algorithms tend to perform worse in terms of the proposed metrics (see additional file 1 for more details).

**Figure 3:**
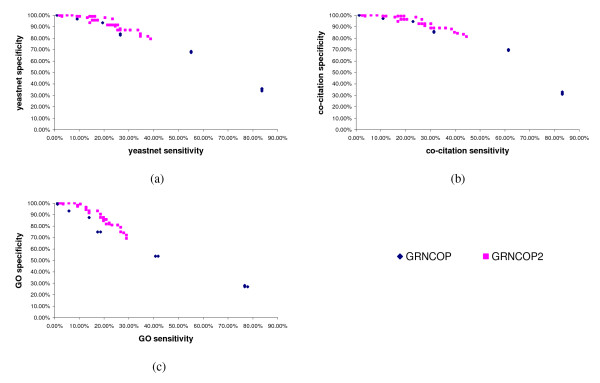
***Sensitivity *and *specificity *of GRNCOP2 and GRNCOP**. The *sensitivity *and *specificity *values achieved by GRNCOP2 and GRNCOP on the three benchmarking sets for the 56 runs. Figure 3a: *sensitivity *vs. *specificity *regarding the Yeastnet set. Figure 3b: *sensitivity *vs. *specificity *regarding the Co-citation set. Figure 3c: *sensitivity *vs. *specificity *regarding the GO set.

Finally, it is also important to analyze the behavior of both algorithms in relation to the *sensitivity *and *specificity *metrics. Figures 3a to 3c depict the *sensitivity *vs. *specificity *of the algorithms on the three benchmarking sets for the 56 runs. It is easy to note that, in general, GRNCOP2 is superior to GRNCOP, since at the same levels of *sensitivity *(*specificity*), the *specificity *(*sensitivity*) achieved by the former is higher. Moreover, although GRNCOP is able to recover almost 80% of the relevant associations in all the cases, this is due to the large number of interactions returned by the algorithm, as it was previously discussed. Therefore, these results show that, in this case of study, GRNCOP2 performs better than GRNCOP, and that the modifications proposed in the new methodology really improve the inference process since the results seem to be more relevant in terms of the *precision, sensitivity, specificity *and *score *metrics.

#### B. Performance of GRNCOP2 vs. rule-based methods

In this section, the performance of GRNCOP2 in terms of the proposed metrics w.r.t. other algorithms described in the literature will be compared. This comparison is limited conforming to the results reported by [7,11,12] due to the unavailability of the algorithms. To make a fair comparison with these methods according to the Rule Consensus Process of GRNCOP2 with an *RCA *parameter of 1, the rules found by the three approaches were filtered in accordance with the accuracy reported. In this way, only the rules which achieved an accuracy of at least 0.75 on the three datasets (cdc15, cdc28 and alpha-factor) were selected for this study. In the case of [11] and [12], the comparison is carried out only with simultaneous rules since the single time-delayed rules in [11] were validated with just one dataset (cdc15) and in [12] the accuracy values were not reported for those rules. Therefore, Table 4 shows the results of the proposed metrics for the simultaneous rules of [11,12] and GRNCOP2 executed on the three datasets with an *Accuracy *of 0.75, a *SCP *of 0.95, an *RCA *of 1 and with **W **= 0. Table 5 shows the same metrics for the rules with time-delays from 1 to 5 of [7] and for GRNCOP2 with the same previous parameterization, except for **W **which was set to 5 and then, the simultaneous rules were removed in order to make the comparison. This parameterization has been established as follows: **W **= 0 denotes that GRNCOP2 will only perform the search of the simultaneous rules; in the case of **W **= 5, it denotes that the search will be carried out upon five units of time-delay; *RCA *= 1 says that the rules must predict well in all the datasets; *SCP *= 0.95 aims to obtain rules of the cases -3,-2, 2 and 3 with high **TP **(**TN**) rates; and the *Accuracy *= 0.75 is intended to represent the same level of accuracy of the other methods, although this is not necessarily true due to the different criteria employed in each algorithm for the evaluation of the rules. Note that the values of the metrics in Table 4 and Table 5 were calculated considering only the pair-wise interaction sets of the genes, as it was previously explained.

**Table 4:** Values of the metrics achieved by GRNCOP2, Soinov *et al. *and Bulashevska and Eils

		GRNCOP2	Soinov *et al*.	Bulashevska and Eils	RANDOM
**Yeastnet**	*precision*	**93.33%**	50.00%	88.89%	51.58%
	*sensitivity*	**14.29%**	3.06%	8.16%	-
	*specificity*	**98.91%**	96.74%	**98.91%**	-
	*score*	**3.04**	1.84	2.77	1.5303384

**Co-citation**	*Precision*	**93.33%**	50.00%	88.89%	43.68%
	*sensitivity*	**16.87%**	3.61%	9.64%	-
	*specificity*	**99.07%**	97.20%	**99.07%**	-
	*score*	**3.26**	1.85	2.84	1.3487118

**GO**	*precision*	**73.33%**	50.00%	55.56%	45.26%
	*sensitivity*	**12.79%**	3.49%	5.81%	-
	*specificity*	96.15%	**97.12%**	96.15%	-

*number of associations*		**15.00**	6.00	9.00	-

Values achieved by GRNCOP2, Soinov *et al. *and Bulashevska and Eils for the proposed metrics, for simultaneous rules at an accuracy level of 0.75. The *precision *and *score *of the random selection is also included. The bolded scores denote the best values.

**Table 5:** Values of the metrics achieved by GRNCOP2 and Li *et al*

		GRNCOP2	Li *et al*.	RANDOM
	*precision*	**100.00%**	**100.00%**	51.58%
**Yeastnet**	*sensitivity*	**10.20%**	5.10%	-
	*specificity*	**100.00%**	**100.00%**	-
	*score*	**3.03**	2.89	1.5303384

	*Precision*	**100.00%**	**100.00%**	43.68%
**Co-citation**	*sensitivity*	**12.05%**	6.02%	-
	*specificity*	**100.00%**	**100.00%**	-
	*score*	**3.37**	2.99	1.3487118

	*precision*	**90.00%**	80.00%	45.26%
**GO**	*sensitivity*	**10.47%**	4.65%	-
	*specificity*	**99.04%**	**99.04%**	-

number of associations		**10.00**	5.00	-

Values achieved by GRNCOP2 and Li *et al. *for the proposed metrics, for rules with time-delays from 1 to 5 at an accuracy level of 0.75. The *precision *and *score *of the random selection is also included. The bolded scores denote the best values.

As shown, GRNCOP2 performs equally or better with this level of accuracy in terms of almost all the proposed metrics. The differences w.r.t. the referential methods are more evident in the case of the simultaneous rules (see Table 4) than in the case of the time-lagged rules (see Table 5). However, in this last scenario, GRNCOP2 is able to recover twice as many relevant interactions (see the *sensitivity *values) as Li *et al. *with the same level of *precision*. Although these results are not conclusive in the determination of the best method since it is limited to only one case of study in one level of accuracy, they provide insight regarding the real performance of the proposed approach. In this sense, these observations clearly indicate that GRNCOP2 is a method capable of inferring relevant interactions with high levels of *precision *that other methods of the literature are unable to find.

#### C. Biological relevance of the results

The rules obtained by GRNCOP2 with an *Accuracy *= 0.75, a *SCP *= 0.95, *RCA *= 1 and **W **= 5, for the cdc15, cdc28 and alpha-factor datasets are summarized in Table 6. The results depicted in this table are in fact the same as those employed in the comparison study of the previous subsection. The only difference lies in that, in this case, the time-delay and the symmetric links of the interactions are maintained, given that the evaluation with the benchmarking sets is not performed. The last three columns indicate interaction relationships that were also inferred by the other methods, using the same three datasets and the same level of accuracy, as it was previously described. Only rules of the cases 1 and -1 were found due to the high *SCP *value employed. Note that multiple time-delays allow for discovery of additional interactions that are not visible in GRNCOP. Also, none of the time-delayed rules found by GRNCOP2 were found by [7], and thus the corresponding column was omitted on Table 6. This fact might be related to the different discretization processes, since in [7] a fixed threshold (zero) was employed to determine the states of all genes in all time-delays.

**Table 6:** Rules inferred by GRNCOP2

						Rule found by		
Rules						GRNCOP	**Soinov et. al**.	Bulashevska and Eils
+/-	*CLB1*	*0*	→	+/-	*CLB2*	*	**-**	**+**
+/-	*CLB1*	*3*	→	+/-	*CLB5*			
+/-	*CLB1*	*3*	→	+/-	*CLB6*			
+/-	*CLB1*	*0*	→	-/+	*CLN2*	*		
+/-	*CLB1*	*0*	→	+/-	*SWI5*	*		
+/-	*CLB2*	*0*	→	+/-	*CDC20*			
+/-	*CLB2*	*0*	→	+/-	*CLB1*	*	*	**+**
+/-	*CLB2*	*3*	→	+/-	*CLB5*			
+/-	*CLB2*	*3*	→	+/-	*CLB6*			
+/-	*CLB2*	*0*	→	-/+	*CLN2*			
+/-	*CLB2*	*0*	→	+/-	*SWI5*	*		
+/-	*CLB5*	*4*	→	+/-	*CLB2*			
+/-	*CLB5*	*0*	→	+/-	*CLB6*	*	**+**	
+/-	*CLB5*	*3*	→	-/+	*CLB6*			
+/-	*CLB6*	*0*	→	-/+	*CLB1*			**+**
+/-	*CLB6*	*0*	→	+/-	*CLB5*	*	**+**	
+/-	*CLB6*	*3*	→	-/+	*CLB5*			
+/-	*CLB6*	*0*	→	+/-	*CLN2*	*		
+/-	*CLB6*	*1*	→	+/-	*CLN2*			
+/-	*CLN1*	*0*	→	+/-	*CLB6*	*		
+/-	*CLN1*	*2*	→	-/+	*CLB6*			
+/-	*CLN1*	*0*	→	+/-	*CLN2*	*		
+/-	*CLN2*	*4*	→	+/-	*CLB2*			
+/-	*CLN2*	*0*	→	+/-	*CLB5*	*		
+/-	*CLN2*	*0*	→	+/-	*CLB6*	*		
+/-	*CLN2*	*2*	→	-/+	*CLB6*			
+/-	*CLN2*	*3*	→	-/+	*CLB6*			
+/-	*SIC1*	*0*	→	+/-	*CLB5*			
+/-	*SWI4*	*0*	→	+/-	*CLB5*			
+/-	*SWI4*	*4*	→	-/+	*CLB5*			
+/-	*SWI5*	*0*	→	+/-	*CDC20*	*		
+/-	*SWI5*	*0*	→	+/-	*CLB2*	*		**+**
+/-	*SWI5*	*3*	→	+/-	*CLB6*			

Rules inferred by GRNCOP2 using 20 cyclin genes of the datasets from Spellman *et al. *(1998). The last three columns indicate whether the rules were found by any of the other methods. The complete rules are represented by an *; + for positive regulatory relationship, and - for the negative regulatory relationship.

The biological relevance of the inferred rules was estimated by analyzing whether such relationships reflected key functional properties relating to the different cell cycle phases: G1, S, G2, M, M/G1. Genes *CLN1 *and *CLN2 *transcribe G1-cyclins, while *CLB5 *and *CLB6 *transcribe B-cyclins. They share a similar expression pattern and attain their highest expression levels during the G1 phase, which can be verified in the analyzed experimental data [36-38]. This knowledge is consistent with the rules: +/-*CLB6 0→ *+/-*CLB5*, +/-*CLB6 0→ *+/-*CLN2*, +/-*CLN2 0→ *+/-*CLB5*, +/-*CLB5 0→ *+/-*CLB6*, +/-*CLN1 0→ *+/-*CLB6*, +/-*CLN2 0→ *+/-*CLB6*, +/-*CLN1 0→ *+/- *CLN2 and +/- CLB6 1→ *+/-*CLN2*. These rules are also consistent with some observations on the partial functional redundancy existing among *CLB5, CLN1 *and *CLN2*, which has been reported by Epstein and Cross [39] and Levine *et al. *[40]. In particular, the short time-delay link indicated by the rule +/-*CLB6 1→ *+/-*CLN2*, detected only by GRNCOP2, can be explained in terms of the progression of the mRNA concentrations of genes *CLB5, CLB6 *and *CLN2 *at the beginning of the yeast cell cycle, as it is detailed in the budding yeast molecular model presented by Chen *et al. *[37].

*CLB1 *and *CLB2 *are specific cyclins of the G2 phase, and there is biological evidence that they are co-expressed in this process [41]. Gene *SWI5 *is a transcription factor whose activation occurs during the G2 phase. These facts justify the following rules: +/-*CLB2 0→ *+/-*CLB1*, +/-*CLB1 0→ *+/-*CLB2*, +/-*CLB1 0→ *+/-*SWI5*, +/-*CLB2 0→ *+/-*SWI5*, +/-*SWI5 0→ *+/-*CLB2*, which are further supported by biological evidence presented by Koranda *et al. *[42]. In particular, the rule +/-*SWI5 0→ *+/-*CLB2 *was only discovered by the algorithm GRNCOP2. Furthermore, the transcription of *SWI5 *is activated later in phase S, and its peak of mRNA concentration occurs during the G2 phase [43]; whereas *CLB6 *is active in phase G1 of the cell cycle. This information is consistent with the time-lagged rule: +/-*SWI5 3→ *+/-*CLB6*.

It is also well know that in budding yeast the G1 cyclins, such as *CLN1 *and *CLN2*, are expressed in G1 and S phases, while mitotic cyclins such as *CLB1 *and *CLB2 *are expressed in G2 and M phases. Amon *et al. *[44] found that the *CLBs *play a central role in the transition from S to G2 phases, showing evidence that *CLBs *repress *CLNs*. This negative regulation of *CLNs *may occur via the transcription factor *SWI4*, because *CLBs *are necessary for G2 repression of SCB-regulated genes like *CLN1 *and *CLN2*. On the other hand, Andrews and Measday [45] present evidence that the Cyclin/CDK complexes (*CDC28*/*CLN1 *and *CDC28*/*CLN2*) regulate *CLB *proteolysis. This data is consistent with the inhibitory relationships inferred between G1- and G2-specific genes: +/-*CLB1 0→ *-/+*CLN2*, +/-*CLB6 0→ *-/+*CLB1 *and +/-*CLB2 0→ *-/+*CLN2*. In particular, the last rule was only inferred by GRNCOP2. The reader is referred to [41,43] and [46] for additional detailed information on the biological relevance of these associations.

With regard to *SIC1*, it is well known that this gene is an inhibitor of *CLB *complexes, and that it is active during the G1 phase - together with *CLB5 *and *CLB6 *- inhibiting *CLB1 *and *CLB2 *[47]. This knowledge validates the new rule: +/-*SIC1 0→ *+/-*CLB5 *inferred by GRNCOP2. *CDC20 *and *SWI5 *are transcribed later in the S/G2 phase [38], which explains the association represented by the rule: +/-*SWI5 0→ *+/-*CDC20*. This rule was not detected by the methods compared with GRNCOP2. Printz *et al. *[48] presented evidence that *CLB2 *stimulates the synthesis of *CDC20*. This feature is captured by the rule: +/-*CLB2 0→ *+/-*CDC20*.

The protein *SWI4 *is a component of the SBF complex, which controls the expression of genes during phase G1 [49]. This is in accord with the activator role of *SWI4 *on the genes expressed in the G1 phase, as represented by the rule: +/-*SWI4 0→ *+/-*CLB5*. These observations offer evidence of the biological relevance of the association rules inferred by GRNCOP2.

**Figure 4:**
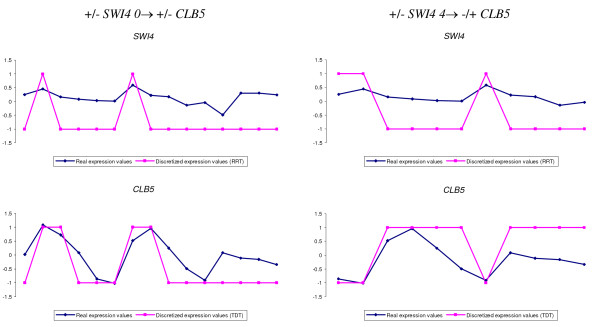
**Discretized values for *SWI4 *and *CLB5 *genes on the cdc15 dataset**. Real and discretized expression profiles of *SWI4 *and *CLB5 *genes with 0 (left) and 4 (right) units of time-delay for the cdc15 dataset.

Finally, the opposite behavior between G1- and G2-specific genes - as it is evidenced by rules obtained from the analysis of simultaneous time-points of the microarray datasets - turns into similar activation patterns when some time-delay is considered, as a consequence of the pattern comparison through the different cellular phases. In other words, if GRNCOP2 matches the behavior of a G1-cycling gene in G1 phase with the behavior of a G2-cycling gene in G2 phase a positive correlation is inferred. This is the case of the following rules: +/-*CLB1 3→ *+/-*CLB5*, +/-*CLB1 3→ *+/-*CLB6*, +/-*CLB2 3→ *+/-*CLB5*, +/-*CLB2 3→ *+/-*CLB6*, +/-*CLB5 4→ *+/-*CLB2 *and +/-*CLN2 4→ *+/-*CLB2*. In a similar way, when GRNCOP2 compares the activation patterns of genes with high expression levels during the G1 phase in contrast with the expression pattern of these same genes during G2 phase, some opposite and logical relationships may emerge: +/-*CLB5 3→ *-/+*CLB6*, +/-*CLB6 3→ *-/+*CLB5*, +/-*CLN1 2→ *-/+*CLB6*, +/-*CLN2 2→ *-/+*CLB6*, +/-*CLN2 3→ *-/+*CLB6 *and +/-*SWI4 4→ *-/+*CLB5*. Take for example the rule +/-*SWI4 4→ *-/+*CLB5 *which has a contradictory interaction with the rule +/-*SWI4 0→ *+/-*CLB5*. Figure 4 shows the real and discretized expression profiles of both genes with 0 (left) and 4 (right) units of time-delay for the cdc15 dataset. As it can be observed, both rules are perfectly inferable from the algorithmic point of view and, a priori, equally probable in biological terms. Thus, in such cases of contradictory interactions, a deeper analysis is required in order to establish the actual relation between the genes. Nonetheless, it is important to note that the inference of these time-lagged contradictory interactions can help in the analysis of the dynamic behavior pattern of activation and repression of the genes along the different cell-cycle phases, and may assist in the identification of the phase transitions in the data.

Apart from the previous analysis, it is necessary to clarify that we do not claim that the rules inferred by GRNCOP2 always represent confident regulatory associations between genes. We think that our extracting-rules approach can be useful for the identification of some promising hypothesis, whose corroboration by biological experiments will always be mandatory in order to obtain curated new knowledge. In addition to this, it should be clear that important known interactions will not be found by GRNCOP2 (and by any other data driven approach) if the microarray data does not have correlations among the genes involved in such relations in the time-lags being analyzed.

### Genome-wide study

The aim of this study is to show the usefulness and capability of GRNCOP2 in genome-wide studies. To account for this, we have applied the proposed algorithm to several microarray time series datasets [16,50-54] for the *Saccharomyces cerevisiae *organism, downloaded from the Gene Expression Omnibus (GEO) database [55] and from some other sources [16]. The complete list of sources is summarized in Table 7.

**Table 7:** List of genome-wide datasets

Microarray time-series dataset	Reference	Sample count
GDS1752_d1	Ronen and Botstein [50]	12
GDS1752_d2		14
GDS2003_d1	Lai *et al. *[51]	15
GDS2003_d2		15
GDS2347	Pramila *et al. *[52]	13
GDS2350_d1	Pramila *et al. *[53]	25
GDS2350_d2		25
GDS759	Sapra *et al. *[54]	24
ELUTRIATION	Spellman *et al. *[16]	14
ALPHA FACTOR		18
CDC15		24
CDC28		17

List of datasets employed in the genome-wide study. Some datasets were separated into two different sets of samples based on the experimental conditions described for each one.

In order to perform rule inferences from these datasets, a few previous steps were performed. Since the list of genes reported in each dataset slightly differs from the other datasets, we have selected those genes that have been measured in all the studies. Moreover, this list was filtered according to those genes of the benchmarking databases described before. This results in a final list of 5245 yeast genes over which this study was focused. Additionally, the samples of some datasets [50,51,53] were separated in two different sets based on the experimental conditions described for each one, resulting in 12 different datasets. The whole set of samples was employed for this analysis. Finally, the missing values were estimated employing a bayesian missing value estimation method [56]. It is necessary to clarify that despite the fact that datasets actually have different sampling rates no normalization of these ratios was performed. Thus, the time-delayed rules must be interpreted as it was previously discussed in the Rule Consensus Process section.

For this analysis, 63 runs of the GRNCOP2 algorithm were performed, which result from the variation of the *Accuracy *parameter from 0.70 to 1 with increments of 0.05 and from the variation of the *RCA *parameter from 0.60 to 1 with increments of 0.05.

**Figure 5:**
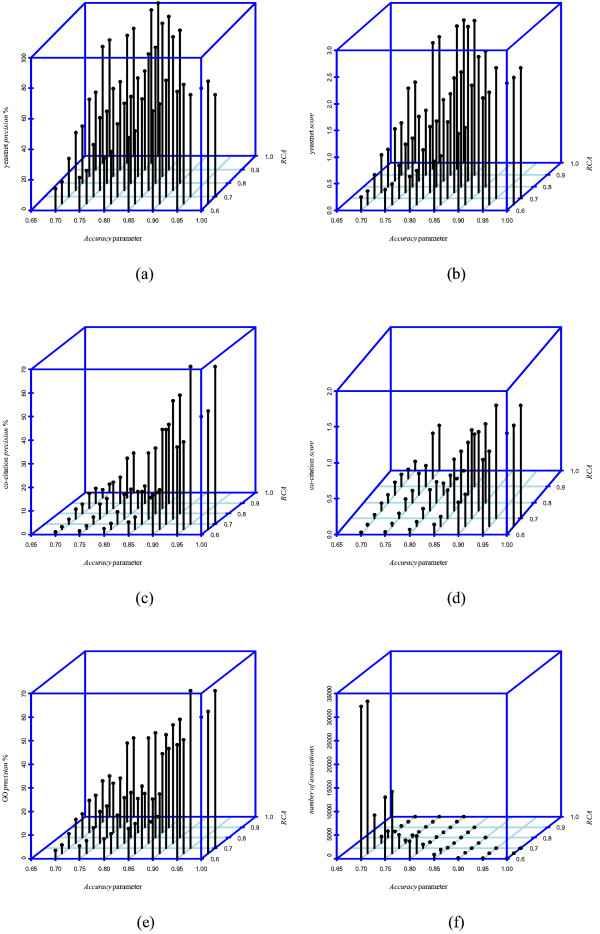
***Precision *and *score *values achieved by GRNCOP2 with different *Accuracy *and *RCA *parameters**. Values of the *precision *and *score *metrics achieved by GRNCOP2 with the *Accuracy *and *RCA *parameters varying from 0.70 to 1 and from 0.60 to 1 respectively, with the *SCP *parameter fixed in 0.95 and with **W **= 4. The *number of associations *is also showed. Figure 5a: yeastnet *precision*. Figure 5b: yeastnet *score*. Figure 5c: co-citation *precision*. Figure 5d: co-citation *score*. Figure 5e: GO *precision*. Figure 5f: *number of associations*.

Additionally, only the rules with a span up to 4 time-delay units (**W **= 4) were inferred since we consider that this value is appropriated (regarding its magnitude) to assess the genome-wide scalability of the algorithm. However, in order to obtain meaningful time-lagged relationship between genes, the researchers are encouraged to follow the recommendation given by (5) considering their hypothesis about the time-delayed regulations that may be present in the experiments. The *SCP *parameter was fixed in 0.95 following the suggested criterion as the objective is to analyze the behavior of the algorithm varying the proportion of datasets that support the rules. Each run took 30 min of execution on a six core processor with 8 gb of ram. As regards the results, Figure 5 shows the *precision *and *score *metrics on the reference sets and the *number of associations *achieved by GRNCOP2 in each run. The points of the upper-right corner of the figures (where the *Accuracy *and the *RCA *parameter get closer to 1) are omitted since the algorithm was unable to obtain any rule with those parameter values. The details of each run are available in the additional file 2.

As it can be observed, as the values of the *Accuracy *and the *RCA *parameters increase, the *precision *and *score *values achieved by the algorithm improve (see Figures 5a to 5e). This is important by the fact that it shows a proper behavior of the Rule Consensus Process and of the objective function (7), since the significance of the set of rules is directly related to the values of those parameters. On the other side, the number of interactions also decreases considerably (see Figure 5f). Even more, if the *sensitivity *metric is considered, GRNCOP2 is only able to recover at most 4.69%, 1.23% and 1.85% of the interactions in the Yeastnet, Co-citation and GO reference sets respectively, values that decrease with the reduction of the *number of associations *(see additional file 2 for details). Although, even though the method seems to achieve a poor performance regarding the *sensitivity *metric, it must be kept in mind the real scale of the genome-wide study performed here, since only 0.70%, 0.20% and 0.45% of all possible gene-pair interactions belong to the Yeastnet, Co-citation and GO benchmark sets respectively. Moreover, these reference sets were obtained employing different sources of information, thus it is not even realistic to expect that they may be recovered using only these microarray data, especially if the information is not present in the gene expression data. Note that in the previous discussion, the *specificity *metric was avoided due to the large amount of TNs that the three reference sets impose, making the algorithm to perform always above the 99% on this measure.

**Figure 6:**
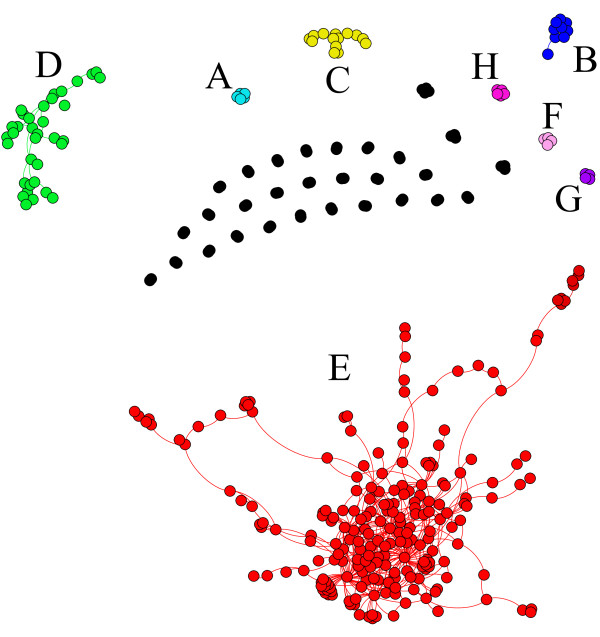
**Reconstructed GRN with *Accuracy *= 0.75, *RCA *= 0.75, *SCP *= 0.95 and W = 4**.

However, the previous analysis says nothing about the biological nature of the GRN obtained from these datasets. Thus, a deeper analysis was performed with the aim of discovering the actual knowledge recovered by GRNCOP2. Figure 6 depicts the GRN obtained in one of the previous runs, which corresponds to an *Accuracy *and *RCA *of 0.75 and 0.75 respectively. This GRN consists of 352 genes and 559 rules (see additional file 3 for details on the rules). The genes were grouped according to their connectivity so as to improve the visualization.

It is easy to conclude that the resulting GRN is not a totally connected graph. Instead, several sub-networks can be visually identified together with other rules that are absolutely disconnected. Therefore, the following question might arise: is it possible for the genes that form each sub-network to be related in some way? This question will be answered performing an ontological analysis over these gene groups. Therefore, the Biological Process, Molecular Function and Cellular Component for the eight largest sub-networks were examined using Onto-Express [57], assuming a hyper-geometric distribution and referencing the calculations by the 5245 genes analyzed. These results are reported in Table 8, together with the values obtained when the whole GRN is considered.

**Table 8:** Ontological analysis for the eight largest sub-networks

	Biological Process			Molecular Function			Celullar Component			*number of genes*
	***annotation***	***percentage***	***corrected*** ***p-value***	***annotation***	***percentage***	***corrected*** ***p-value***	***annotation***	***percentage***	***corrected*** ***p-value***	

**A**	**translation**	**100%**	**0**	**structural constituent of ribosome**	**100%**	**0**	**ribosome**	**100%**	**0**	5
**B**	**chromatin assemby or disassembly**	**88.89%**	**0**	**DNA binding**	**88.89%**	**0**	**nucleosome**	**88.89%**	**0**	9
**C**	**cell cycle**	**50%**	**5.80E-04**	DNA binding	35.71%	0.06779	nucleus	71.43%	0.04154	14
**D**	**DNA replication**	**37.14%**	**0**	**DNA binding**	**40%**	**0**	**nucleus**	**71.43%**	**3.40E-04**	35
**E**	**ribosome biogenesis**	**53.74%**	**0**	molecular function	38.79%	0.02142	**nucleus**	**62.62%**	**0**	214
**F**	**cell division**	**75.00%**	**0.00247**	molecular function	50.00%	0.35995	cytoplasm	50.00%	0.73076	4
**G**	**methionine biosynthetic process**	**100.00%**	**0**	transferase activity	75.00%	0.01018	**cytoplasm**	**100.00%**	**0**	4
**H**	biological process	60.00%	0.18427	**nucleic acid binding**	**80.00%**	**3.00E-05**	cellular component	60.00%	0.04812	5

all	**ribosome biogenesis**	**35.51%**	**0**	molecular function	33.24%	0.31227	**nucleus**	**57.39%**	**0**	352

The eight largest sub-networks, with their respective ontological enrichment, for the Biological Process, Molecular Function and Cellular Component categories. The *annotation *column denotes the most common annotation for the genes in the sub-network, whereas the *percentage *is the percentage of genes w.r.t. the *number of genes *in the sub-network that receives such annotation. The *corrected p-value *is the statistical significance of the annotation. Finally, the bolded categories and scores remark the cases where the annotation was statistically significant at an α level of 0.01.

As shown in Table 8, all of the sub-networks achieved a relatively high non-trivial ontological enrichment in at least one of the categories, and these results are statistically significant at an α level of 0.01. Moreover, the proportion of gene enrichment of each group is higher than the proportion of gene enrichment of the whole GRN. These results demonstrate that the genes of each sub-network are highly related to each other, relations that are also established directly or indirectly through the rules discovered by GRNCOP2.

## Conclusions

In this paper, a model-free combinatorial optimization algorithm designed for the inference of putative GRNs called GRNCOP2 was presented. Although the basic ideas behind GRNCOP remain in GRNCOP2 (that is, the adaptive regulation thresholds and the combinatorial optimization of rules' classifiers), the method presented in this article is a new algorithm that constitutes a relevant evolution of the previous method due to the challenges that impose the proposed improvements. The new algorithm incorporates novel features such as inference of rules with multiple time-delays and on an unlimited number of time series datasets, and improvements over the whole inference process. This last feature was demonstrated by the fact that the results achieved by GRNCOP2 are significantly better than those obtained by the previous version. As well, the relevance of the new method became more evident since the scores achieved by GRNCOP2 were superior to those obtained by other related algorithms in terms of the proposed metrics. In addition, the relationships inferred by GRNCOP2 proved to be biologically relevant. Even more, it was able to obtain new potential interactions between genes, consistent with previous biological knowledge, that were not discovered by the other methods.

Additionally, the ability of GRNCOP2 to perform genome-wide studies was assessed. In this regard, a study was performed over several genome-wide time series datasets, for which the proper functioning of the algorithm in terms of the proposed metrics was discussed. Also, with the realization of an ontological analysis it has been showed that the results were significant in biological terms, since the genes of the discovered sub-networks were found to be highly related in statistical terms.

However, this study does not claim that the data-driven machine learning approach proposed in this paper is sufficient to infer biologically meaningful regulatory networks. Nevertheless, this tool offers significant evidence necessary to aid scientists in exploring and identifying biologically relevant associations, whose assessment by biological experiments is obligatory in order to achieve curated new knowledge.

## Authors' contributions

CAG designed and programming the algorithm GRNCOP2, conducted the computational experiments, proposed and contrasted performance metrics among the different methods, and drafted the manuscript. JAC participated in the design and coordination of the study and strongly contributed to improving the draft of the manuscript. IP is author of GRNCOP (ancestor of GRNCOP2), designed and coordinated the study, and performed and wrote the biological relevance analysis of the association rules inferred for cycling yeast genes. All authors read and approved the final manuscript.

## Supplementary Material

Additional file 1 **Individual values of the metrics for each run of GRNCOP2 and GRNCOP**. The individual results of each run of both algorithms measured in terms of *precision, sensitivity, specificity *and *score *metrics regarding the reference sets are depicted in the table of the file. This is the information used in the comparison of GRNCOP2 and GRNCOP in the subsection A of the comparative study. Click here for file

Additional file 2 **Individual values of the metrics for each run of GRNCOP2 in the genome-wide study**. The individual results of each run of the GRNCOP2 algorithm measured in terms of *precision, sensitivity, specificity *and *score *metrics regarding the reference sets are depicted in the table of the file. This is the information used in the discussion about the performance of GRNCOP2 in the genome-wide study. Click here for file

Additional file 3 **Rules of the GRN corresponding to Figure 6**. The rules obtained for the genome-wide study with an *Accuracy, RCA, SCP*, and **W **of 0.75, 0.75, 0.95 and 4 respectively, is reported in a Table separated value file. The last two columns indicate the *Accuracy *and *RCA *achieved for each rule. Click here for file
